# Acute Hypoxia Induces Cardiomyocyte Autophagy via SIRT5/mTOR/ULK1 Pathway

**DOI:** 10.1155/bmri/8867024

**Published:** 2026-10-09

**Authors:** Yunle Wang, Hongqi Wei, Yuxuan Wu, Yang Luo

**Affiliations:** ^1^ Geriatrics Department Shanghai Health and Medical Center Wuxi Jiangsu China; ^2^ Otorhinolaryngology Department Suzhou Dushu Lake Hospital Suzhou Jiangsu China; ^3^ Medical College Suzhou University Suzhou Jiangsu China usts.edu.cn; ^4^ Radiology Department Shanghai Health and Medical Center Wuxi Jiangsu China

**Keywords:** acute myocardial infarction, autophagy, hypoxia, mitochondrial, mTOR, SIRT5

## Abstract

**Objective:**

The aim of this study is to investigate the expression of SIRT5 in acute myocardial hypoxia and to further explore its function during this pathological process.

**Material and Methods:**

We analyzed the microarray dataset GSE27975 derived from the Gene Expression Omnibus (GEO) database and identified differentially expressed genes (DEGs) between hypoxic and normoxic cardiomyocytes. We divided C57BL/6 J mice into two groups: the sham group and the acute myocardial infarction (MI) group. Heart functions were detected by echocardiogram, and heart tissue was harvested on the 3rd day after MI for the detection of autophagy and SIRT5 expression. To further study the mechanism of Sirt5 in hypoxic cardiomyocytes, we divided H9C2 cells into four groups: the control group, the hypoxia group, the Sirt5‐knockdown group and the Sirt5‐knockdown + hypoxia group and detected Sirt5 expression, cell autophagy and potential pathway.

**Results:**

A total of 156 genes were highly expressed, and 185 genes were expressed at low levels between hypoxic and normoxic cardiomyocytes. SIRT5 was expressed at low levels in the hypoxia group. In vivo, we generated the MI mouse model, and heart function was decreased. The expression of SIRT5 in heart tissue was decreased after MI, whereas the autophagy level was increased. In vitro, after we knocked down Sirt5 in H9C2 cardiomyocytes, autophagy was significantly increased. Meanwhile the expression of p‐mTOR and p‐ULK1 changed, whereas both mitochondrial membrane potential and respiratory capacity were maintained.

**Conclusion:**

Acute hypoxia reduces SIRT5 expression may be associated with cardiomyocyte autophagy. Knockdown Sirt5 in H9C2s could activate autophagy via mTOR/ULK1 pathway and preserve mitochondrial function.

## 1. Background

Acute myocardial infarction (MI) is one of the leading causes of death and leads to over 20% of deaths worldwide every year [1]. After acute MI, fibrosis fills the infarct areas due to the inability of the heart tissue to regain new myocytes and left ventricular (LV) myocardial remodeling occurs [2]. This is the most common mechanism that leads to functional decompensation and heart failure [3]. As the prognosis of heart failure is poor, exploring efficient measurements of therapy to prevent myocardial loss after acute ischemia is necessary.

Autophagy is a process that supports cell homeostasis and metabolism, which is induced by unfavorable conditions such as nutrient deprivation and hypoxia [4]. In the heart tissue, autophagy is mainly stimulated by myocardial ischemia [5]. At the very early stage of ischemia, cardiomyocytes would undergo adaptable autophagy, which prevents apoptosis, and inhibition of this process would increase cell death in vitro and impair heart dysfunction in vivo [6, 7].

The main molecular mechanisms of autophagy induction are the PI3K/AKT and AMPK pathways. In mammalian cells, mTOR activation could inhibit the AMPK pathway, whereas mTOR inhibition could activate autophagy [8, 9]. Recently, one of the sirtuin (SIRT) family NAD+ ‐dependent Lys deacetylases, SIRT5, was shown to regulate key biological processes in mammals and induce autophagy in tumor cells [10, 11]. Nevertheless, whether SIRT5 participates in modulating autophagy during myocardial ischemia remains largely unknown. We detected the expression of SIRT5 after myocardial ischemia both in vivo and in vitro and then investigated its regulation of myocardial autophagy.

## 2. Methods

### 2.1. Data Sources

Gene expression data of GSE27975 are downloaded from the NCBI Gene Expression Omnibus (GEO: http://www.ncbi.nlm.nih.gov/geo).The expression profiling arrays were generated using the GPL1261 platform (Affymetrix Mouse Genome 430 2.0 Array). A total of 6 HL‐1 cardiomyocyte samples in this database, including three from hypoxia group and three from normoxia group, were analyzed.

### 2.2. Identification of Differentially Expressed Genes (DEGs)

Platform files and genetic matrix files were downloaded to eliminate errors and make the hypoxia group comparable with the normoxia group, so the obtained data were standardized. The estimation package is based on the KNN (k‐nearest neighbor) algorithm and is used to fill the missing values. After that, the probes were converted into gene symbols based on the annotation platform file. If there were multiple probes corresponding to a gene, the average was taken as the final value. If the probe without a gene symbol was removed, the normalized between‐array function in the limma package was applied to standardize the data. The expression data were log2 transformed, and the limma functional package in R software was used to compare gene expression in hypoxia and normoxia samples to identify DEGs. The screening criteria for DEGs were *p* value < 0.01 and |log_2_fold change (FC)| ≥ 2.

### 2.3. Mouse Model of MI

Briefly, mice were intraperitoneally anaesthetized with pentobarbital sodium (50 mg/kg). After anesthesia, mice were fixed in the supine position by tying the legs and the upper jaw. The electrocardiogram (ECG), heart rate and respiratory rate were continuously monitored. They were intubated and ventilated to perform left thoracotomy and LAD coronary artery ligation. Ischemia was confirmed by myocardial blanching and ECG ST‐segment elevation. Sham operations were carried out by the same method but without the ligation of the LAD. Then, we administered an intramuscular injection of penicillin. After restoring spontaneous breathing, endotracheal intubation was pulled out, and then mice were placed on a warm blanket waiting for their revival. All animal experimental protocols were approved by the Soochow University Animal Care Committee and research protocols were conducted in accordance with the animal behavioral guidelines, using approved protocols from the institutional animal care committee (#202203A0115).

### 2.4. Echocardiography Measurement

High‐frequency ultrasound system Vevo2100 (VisuaSonic Inc, Toronto, Ontario, Canada) with a 30 MHz central frequency scan head was used to estimate heart function [12]. Before and on the 3rd day after MI, anesthesia was performed with 1%–2% isoflurane vapor. The echocardiographic images in short and long axes were obtained by two experienced and independent operators blinded to the experiments while on the condition of monitoring heart rate, respiration rate and ECG. The left ventricular ejection fraction (LVEF) and left ventricular fractional shortening (LVFS) were calculated by parameters including left ventricular end systolic diameter (LVESd), left ventricular end‐diastolic diameter (LVEDd), left ventricular posterior wall end‐systolic thickness (LVPWTs), left ventricular posterior wall end‐diastolic thickness (LVPWTd), interventricular septum end‐systolic thickness (IVSd), and interventricular septum end‐diastolic thickness (IVSs).

### 2.5. Immunohistochemistry Staining

To evaluate the morphological changes and the extent of autophagy, the hearts were harvested on the 3rd day after MI, washed in phosphate‐buffered saline (PBS), fixed in 4% paraformaldehyde and embedded in paraffin. Each heart was cut into 4‐mm‐thick sections. After dewaxing, hydration and antigen repair, LC3 antibody was incubated for 2 h, washed three times with PBS and incubated in biotin‐labeled secondary antibody for 10 min. We restained it with hematoxylin stain and washed in running water for 1 min. Subvalvular regions were regarded as film reading areas in each group, and the staining was observed under a light microscope.

### 2.6. Cell Hypoxia

H9C2 cell hypoxia was assessed using an AnaeroPack‐Anaero (MGC, Japan) and anaerobic jar. After 30 min, the pack consumed all the oxygen and produced an equivalent carbon dioxide; then the mixture gasses in the jar were CO2 and N2. Anaerobic indicator was used in the anaerobic jar, and it showed the concentration of oxygen was below 0.1% during the whole hypoxia process. The culture medium was changed to DMEM (Gibco, United States) without glucose and serum before hypoxia. H9C2 cells, anaerobic indicator and AnaeroPack were locked in anaerobic jars. The whole anoxic process was conducted at 37°C for 6 h.

### 2.7. mRFP‐GFP‐LC3 Adenovirus Infection

H9C2 cells were plated on slides in 24‐well plates and reached 50%–70% confluence. mRFP‐GFP‐LC3 adenoviral (HanBio, Shanghai, China) infection was performed according to the manufacturer′s instructions. The RFP and GFP fusion points represent the autophagosomes, whereas the RFP points represent the autolysosomes. H9C2 cells were incubated in growth medium with the adenoviruses at an MOI of 100 for 2 h at 37°C and then replaced with new medium for another 24 h at 37°C. After infection, cells were subjected to different treatments according to different groups. Cells were observed under a fluorescence microscope (Olympus BX 51, Tokyo, Japan). Autophagic flux was determined by evaluating the number of GFP, RFP, and merged points (points/cell were counted).

### 2.8. SIRT5 siRNA Transfection

Delivering plasmid DNA constructs (SIRT5 siRNA) into cells using TurboFectin 8.0 (Origene Technologies, TF81001) generated stable clones. Cells were seeded on a 24‐well plate. TurboFectin reagents were mixed with serum‐free DMEM at room temperature. Then, plasmid DNA was added to the TurboFectin‐containing media and incubated at room temperature for 30 min. After that, the mixtures were added to the plate with cells. The selection of stable silenced clones was started 24 h later with the addition of 500 *μg*/mL geneticin (Sigma–Aldrich, A1720).

### 2.9. Western Blotting Analysis

Total protein was obtained from LV myocardial tissues on the 3rd day after MI or sham operation. H9C2 cells were lysed in lysis buffer containing phosphatase inhibitor and protease inhibitor. After centrifugation, the samples were sonicated and denatured by heating. A total of 20 *μg* protein lysates were separated on 6% and 12% SDS–PAGE gels and transferred onto nitrocellulose membranes. After the membranes were blocked, they were incubated overnight at 4°C with primary antibodies, including anti‐SIRT5(Cat. No. 8782), anti‐ATG5(Cat. No.12994), anti‐BECLIN1(Cat. No. 3738), anti‐mTOR(Cat. No. 2972), anti–p‐mTOR(Cat. No. 2971), anti‐ULK1(Cat. No. 8054), and anti‐p‐ULK1(Cat. No. 14202). All the above mentioned antibodies were purchased from Cell Signaling Technology (Danvers, Massachusetts, United States). The membranes were then incubated with HRP‐conjugated secondary antibodies. The antigene antibody complexes were detected by SuperSignal ECL kit in western blotting detection system. The results were expressed as density values normalized to GAPDH.

### 2.10. JC‐1

Mitochondrial function was detected by mitochondrial membrane potential (△*Ψ*m) by JC‐1 kit (Beyotime Biotechnology). H9C2s were seeded in 6‐well plate and allowed to grow for 48 h. After treatment under different conditions as indicated cells were collected and suspended in DMEM medium. After dying with dyeing working fluid for 20 min at 37°C, cells were taken photos under fluorescence microscopy.

### 2.11. Reactive Oxygen Species (ROS) Measurement

Intracellular ROS levels were determined using the ROS Assay Kit (KGA7308‐100; KeyGen BioTECH, China) following established protocols with modifications. Briefly, cells were harvested at 80% confluence and seeded in 96‐well black plates (Corning, United States) at a density of 1 × 10^4^ cells/well. After treatment, cells were incubated with 10 *μ*M DCFH‐DA in serum‐free medium at 37°C for 30 min in the dark. Excess probe was removed by three washes with PBS (pH 7.4). For microscopy analysis, cells were imaged using a fluorescence microscope equipped with FITC filters.

### 2.12. Statistical Analysis

Data are expressed as the mean ± standard error of the mean (SEM), and a *p* value of 0.05 was considered statistically significant. Differences were determined using two‐tailed Student′s *t* test or one‐way ANOVA. All statistical analyses were conducted using SPSS 27.0 or GraphPad Prism 5.

## 3. Results

### 3.1. Identification of DEGs

We identified 341 DEGs between hypoxic and normoxic cardiomyocytes in the GSE27975 dataset, which were statistically significant (adjusted *p* < 0.05, |logFC| ≥ 2). Among these, 156 genes were highly expressed, and 185 genes were expressed at low levels (Figure 1C). We chose the Top 54 upregulated genes and 55 downregulated genes (Figure 1C). The results identified the DEGs.

**Figure 1:**
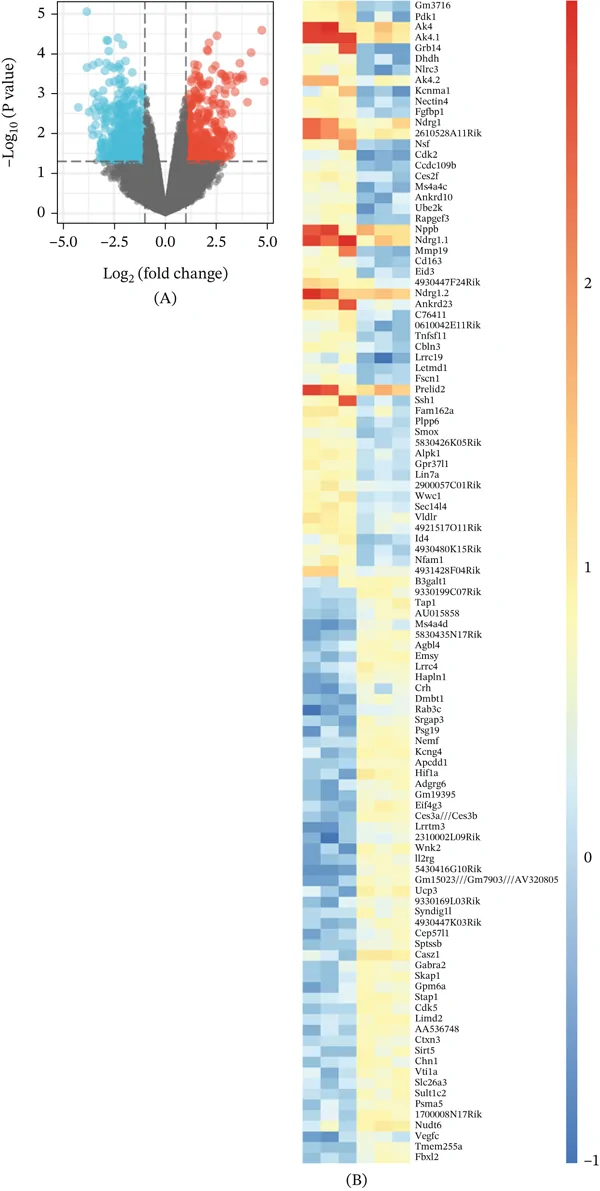
Differential genes between the hypoxia and normoxia cell groups. (A) Volcano plot. (B) Heatmap.

### 3.2. SIRT5 Expression Decreased in the Myocardium After Infarction

On the 3rd day after MI, the representative echocardiograms of MI mice showed that the LV anterior wall movement was weakened (Figure 2A). LVEF and LVFS were significantly lower than those in the sham group (*p* < 0.05, Figure 2B,C). Western blot results showed that SIRT5 expression was decreased in heart tissue at 3rd after MI (*p* < 0.05, Figure 2D,E).

**Figure 2:**
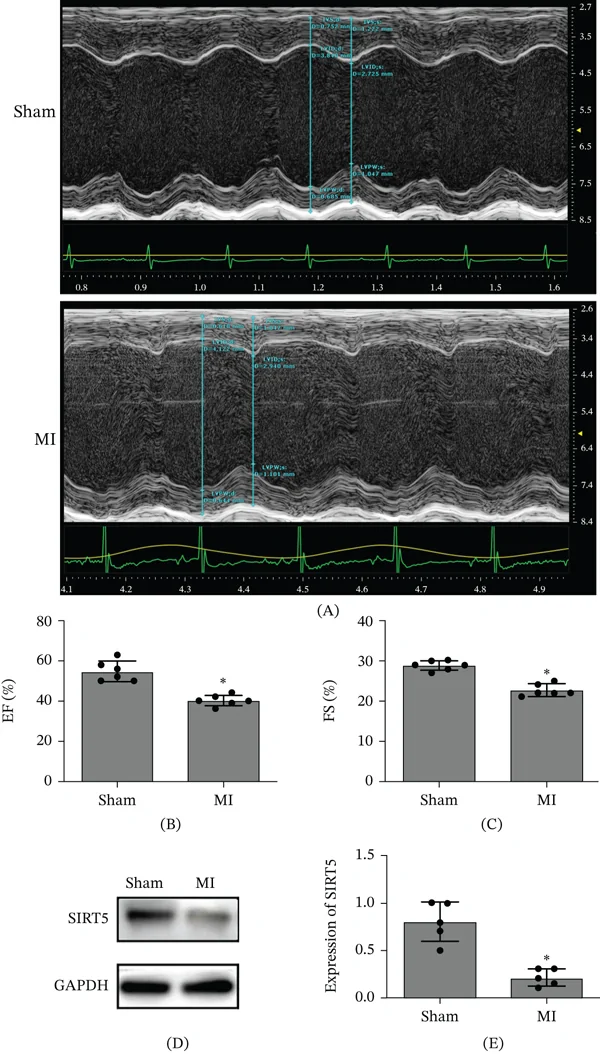
Detection of SIRT5 expression. (A) Echocardiography of the sham and MI mouse groups. (B) Statistics of EF in the sham and MI groups after treatment. (C) Statistics of FS in the sham and MI groups after treatment. (D) Western blot of SIRT5 expression. (E) Densitometry ratio of the expression of SIRT5. The asterisk ( ^∗^) indicates*p* < 0.05compared with the sham group. Sample number is 6 per group.

### 3.3. Hypoxia Increased Myocardial Autophagy Both In Vivo and In Vitro

On the 3rd day after MI, immunohistochemistry demonstrated a significantly increased in LC3 expression in the myocardium in the MI group compared with the sham group (Figure 3A,B). The expression of ATG5 was higher in the MI tissue than sham (Figure 3C,D). In vitro RFP‐GFP‐LC3 adenovirus infection hypoxia stimulated H9C2 autophagy, and both autophagosomes (yellow points) and autolysosomes (red points) were increased after hypoxia (Figure 3E,F). The expression of ATG5 was a significantly increased in the hypoxia group than in the control group (Figure 3G,H).

**Figure 3:**
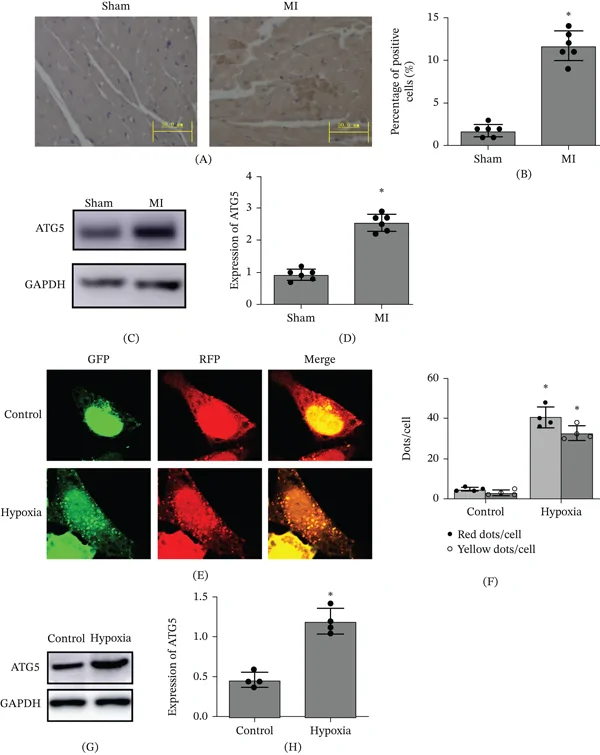
Detection the level of autophagy. (A) Immunohistochemistry of LC3 in the heart of the sham and MI groups. (B) Percentage of LC3‐positive cells in each group. (C) Western blot of ATG5 expression in myocardium. (D) Densitometry ratio of the expression of ATG5. (E) GFP‐RFP‐LC3 fluorescence staining in control and hypoxic cell groups. Scale bar: 5 *μ*m. (F) Statistics of autophagosome and autolysosome fluorescent points. (G) Western blot of ATG5 expression. (H) Densitometry ratio of the expression of ATG5. The asterisk ( ^∗^) indicates*p* < 0.05compared with the hypoxia group. Sample number is 6 per group (A, B, C, D). Independent replicates 4 times per group (E, F, G, H).

### 3.4. SIRT5 Knockdown Increased Myocardial Autophagy Levels

H9C2 cells were treated with SIRT5 siRNA and then divided into four groups: control, hypoxia, SIRT5‐knockdown, and SIRT5‐knockdown + hypoxia. Western blot analysis indicated that the expression of SIRT5 was knocked down by siRNA, and there was no difference between the normoxia and hypoxia groups (Figure 4A,B). ATG5 and BECLIN1 expression was increased after treatment with SIRT5 siRNA. However, there was no significant difference between the SIRT5‐knockdown and SIRT5‐knockdown + hypoxia groups (Figure 4A, C, and D).

**Figure 4:**
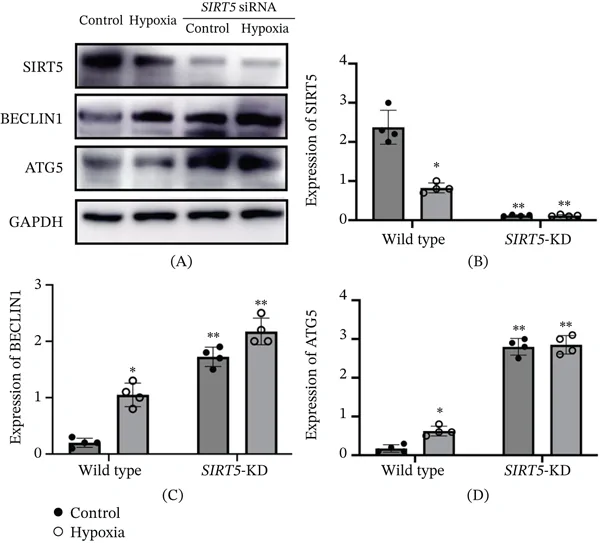
Detection of autophagy after knockdown Sirt5 in H9C2 cardiomyocyte. (A) Western blot of SIRT5, BECLIN1, ATG5 expression of control, hypoxia, Sirt5‐knockdown, and Sirt5‐knockdown + hypoxia groups. (B) Densitometry ratio of the expression of SIRT5. (C) Densitometry ratio of the expression of BECLIN1. (D) Densitometry ratio of the expression of ATG5. The asterisk ( ^∗^) indicates*p* < 0.05compared with the hypoxia group; ( ^∗∗^) indicates*p* < 0.01compared with the hypoxia group. Independent replicates 4 times per group.

### 3.5. SIRT5 Knockdown Regulated Autophagy via mTOR/ULK1 Pathway and Preserved Mitochondrial Function

JC‐1 detection results showed that mitochondrial membrane potential decreased after hypoxia as the mitochondrial membrane RFP/GFP decreased, whereas SIRT5 knockdown preserved mitochondrial membrane potential. ROS assay showed that ROS concentration increased after hypoxia occurred and maintained at a lower level in SIRT5‐knockdown groups, even after hypoxia occurred. These results showed that SIRT5 knockdown protected mitochondrial function after hypoxia in H9C2s (Figure 5A–D). The expression of phosphorylated‐mTOR (p‐mTOR) decreased after hypoxia and SIRT5 knockdown exacerbated this change. After SIRT5 knockdown p‐mTOR expression decreased while after hypoxia it decreased significantly (Figure 5E,F). The expression of phosphorylated‐ULK1 (p‐ULK1) increased slightly after hypoxia, whereas in the SIRT5‐knockdown group, p‐ULK1 increased significantly after hypoxia (Figure 5G,H). These results showed that SIRT5 regulated cell autophagy via mTOR/ULK1 pathway under hypoxic conditions.

**Figure 5:**
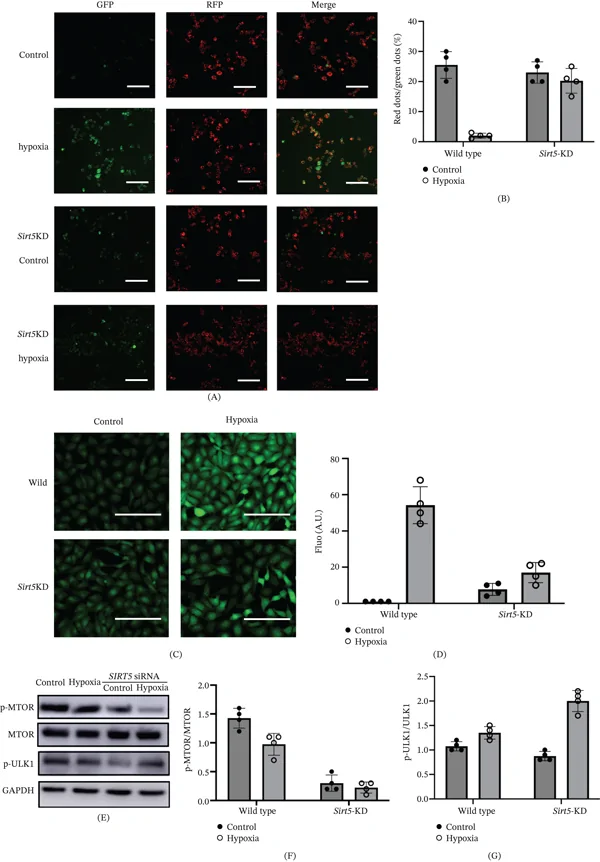
Detection of mitochondrial membrane potential and mTOR/ULK expression after knockdown Sirt5 in H9C2 cardiomyocyte. (A) Fluorescence microscopy for JC‐1 staining of control, hypoxia, Sirt5‐knockdown and Sirt5‐knockdown + hypoxia groups. Scale bar: 20 *μ*m. (B) Statistical of the ratio of RFP/GFP in each group. (C) ROS analysis in each group. Scale bar: 20 *μ*m. (D) Statistical evaluation of the fluorescence intensity. (E) Western blot of mTOR, p‐mTOR, p‐ULK1, GAPDH expression in each group. (F) Densitometry ratio of p‐mTOR/mTOR. (G) Densitometry ratio of p‐ULK1/ULK1. The asterisk ( ^∗^) indicates*p* < 0.05compared with the hypoxia group. Independent replicates 4 times per group.

## 4. Discussion

Adaptive responses to hypoxia exhibit strong tissue specificity. In high‐altitude placentas, RNA editing serves as an important posttranscriptional modulator for long‐term hypoxic adaptation [13]. In this study, we showed that acute MI could attenuate the expression of SIRT5 in heart tissue, and this change may be associated with the regulation of cell autophagy.

A previous study showed that the autophagy process enhancement in the early stage of MI could decrease heart infarction size, reduce adverse ventricular remodeling and preserve heart function [14, 15]. The NAD+ ‐dependent superfamily of proteins called SIRTs are enzymes which involved in posttranslational protein modification in several pathological and physiological processes, including stress resistance (i.e., hypoxia and inflammation), energy control and aging [12, 16]. Initially described as a mitochondrial SIRT, SIRT5 was reported to regulate different metabolic pathways, including fatty acid oxidation, glycolysis, and urea cycle [17]. The study showed that SIRT5 increased in heart after intermittent hypoxia, or transverse aortic constriction, which was opposite to the result we analyzed through the GSE27975 dataset [18, 19]. This is because acute hypoxia causes a totally different pathological change from long‐term hypoxia or heart overload, which has been proved by our early study [7, 20]. In this study, we discovered that SIRT5 expression decreased after acute hypoxia both in vitro and in vivo, which indicated that SIRT5 is a key molecule for sensing oxygen environment that has been proved in hepatic tissue [21]. Knockdown SIRT5 could also increase autophagy in H9C2s after acute hypoxia.

Consistent with the systematic verification from in vitro and in vivo experiments in the present study, acute MI and acute hypoxia stimulation could significantly inhibit the expression level of SIRT5 in cardiac tissues and cardiomyocytes, accompanied by a remarkable activation of cellular autophagy. These findings firmly confirm the close correlation between SIRT5 downregulation and enhanced autophagic activity in the pathological process of myocardial ischemic injury. This showed that SIRT5 was associated with cardiomyocytes autophagy under hypoxia condition.

Several studies have investigated the mechanisms by which SIRT5 regulates autophagy; some studies found out that SIRT5 regulated autophagy via mTOR pathway [22, 23], whereas some discovered that it regulated autophagy via non‐mTOR pathway [10]. ULK1 is a key molecule in regulating autophagy in AMPK/mTOR pathway [24, 25]. Under starvation, AMPK would promote autophagy by directly activating ULK1, whereas high mTOR expression would prevent ULK1 from phosphorylation. In our study, we found out that after acute hypoxia, SIRT5 knockdown decreased mTOR phosphorylation and increased ULK1 activity. These results confirmed that SIRT5 decline was associated with the regulation of hypoxia‐induced autophagy via mTOR/ULK1 pathway. It is worth noting that knocking down SIRT5 did not seem to have an additive effect on autophagy proteins like ATG5 under hypoxic conditions. We guess this may be because SIRT5 regulated the upstream molecules of autophagy‐related pathways.

Studies have shown that after hypoxia, cardiomyocyte mitophagy is one of the major types of autophagy [20, 26]. Although MI occurred, mitophagy increased to remove damaged mitochondria to maintain mitochondrial function and to reduce cell oxidative stress. SIRT5 located on mitochondrial membrane. In mouse embryonic fibroblasts, SIRT5 deletion blunted mitochondrial elongation, resulting in increased mitophagy [23]. SIRT5 activation in breast cancer cells prevents autophagy and mitophagy processes, which impair cell proliferation and accumulate oxidated stress injury [22]. To confirm the effect of SIRT5 on mitochondrial function after hypoxia occurred, we detected the mitochondrial membrane potential and ROS concentration in wild and SIRT5‐knockdown cell groups under acute hypoxia conditions. Results showed that mitochondrial function was preserved in SIRT5‐knockdown group while acute hypoxia occurred. This showed that SIRT5 was important in mitochondrial function in cardiomyocytes while acute hypoxia occurred, which may be associated with the regulation of autophagy.

This study has several limitations that should be acknowledged. First, H9C2 cells are immature embryonic rat ventricular cells with mixed cardiac and skeletal muscle phenotypes and cannot fully recapitulate the characteristics of mature adult cardiomyocytes, which may limit the extrapolation of our findings to adult MI conditions. Second, the in vivo LAD ligation model induced complex myocardial ischemia characterized by hypoxia, nutrient deprivation and metabolite accumulation, rather than isolated pure hypoxia, indicating that the in vivo results reflect comprehensive ischemic injury rather than simple hypoxic damage, which was the same in vitro. In vitro hypoxic intervention was combined with glucose and serum deprivation, so the independent effects of nutrient deficiency and hypoxia on autophagy induction could not be differentiated in the present study. Finally, only gene knockdown manipulation was performed without complementary rescue experiments, which is insufficient to fully confirm the causal relationship of the targeted signaling pathway.

## 5. Conclusion

Acute hypoxia could reduce myocardial SIRT5 expression both in vivo and in vitro, which may be associated with cell autophagy. Knockdown SIRT5 in H9C2s could activate autophagy via mTOR/ULK1 pathway and preserve mitochondrial function.

## Author Contributions

Yuxuan Wu designed this study and did H9C2 cardiomyocyte experiments. Yunle Wang did the animal experiments. Yunle Wang and Yang Luo wrote the manuscript. Yang Luo revised the manuscript. Hongqi Wei did the statistical analysis.

## Funding

This study was supported by the NSFC Young Fund (81900312); Shanghai “Rising Stars of Medical Talents” Youth Development Program (SHWSRS (2023)_062); and the Shanghai Public Health Research Special Program (2026GKM18).

## Ethics Statement

All animal experimental protocols were approved by the Soochow University Animal Care Committee and research protocols were conducted in accordance with the animal behavioral guidelines, using approved protocols from the institutional animal care committee (#202203A0115).

## Consent

The authors have nothing to report.

## Conflicts of Interest

The authors declare no conflicts of interest.

## Data Availability

The data are available on request from the authors.
